# Fetal Sirenomelia Associated with an Abdominal Cyst Originating from a Saccular Cloaca

**DOI:** 10.1155/2018/7513287

**Published:** 2018-03-07

**Authors:** Yui Kinjo, Hitoshi Masamoto, Hayase Nitta, Tadatsugu Kinjo, Tomoko Tamaki, Naoki Yoshimi, Yoichi Aoki

**Affiliations:** ^1^Department of Obstetrics and Gynecology, Graduate School of Medicine, University of the Ryukyus, Okinawa, Japan; ^2^Department of Pathology and Oncology, Graduate School of Medicine, University of the Ryukyus, Okinawa, Japan

## Abstract

A 40-year-old pregnant woman presented with a fetal abdominal cyst and oligohydramnios. Color Doppler scan revealed a single blood vessel from the fetal aorta into a single umbilical artery. Severe oligohydramnios limited ultrasonographic evaluation of the fetal lower limbs, kidneys, or bladder. The pregnancy was terminated; the fetus showed fused lower limbs, bulging abdomen, and absent external genitalia and was diagnosed with type III sirenomelia. On autopsy, no normal bladder was observed, but duodenal atresia, anorectal atresia, and right renal agenesis were found. An intra-abdominal cyst, diagnosed histologically as a saccular cloaca, occupied the abdominal cavity. Ultrasonographic diagnosis of fetal sirenomelia is difficult due to poor depiction of the lower limbs. A vitelline artery leading to a single umbilical artery and a fetal abdominal cyst occupying most of the abdominal cavity are considered fetal sirenomelia associated with large defects of the gastrointestinal and genitourinary tracts.

## 1. Introduction

Sirenomelia is a rare and lethal congenital anomaly characterized by fusion of the lower limbs and additional malformations, including renal agenesis, anorectal atresia, oligohydramnios, and a single umbilical artery. Most infants die within one or two days after birth. Two main pathogenic hypotheses have been proposed for sirenomelia. One is defective blastogenesis hypothesis: the result of a defect in the development of caudal mesoderm during the gastrulation stage occurring in the third week of gestation, and the other is the vascular steal theory: a persistent vitelline artery and abnormalities of the abdominal vasculature lead to a deficient blood flow and nutrient supply to the caudal part of the embryo [1–3].

We experienced a rare case of fetal sirenomelia associated with an abdominal cyst and a single umbilical artery.

## 2. Case Presentation

**Figure 1:**
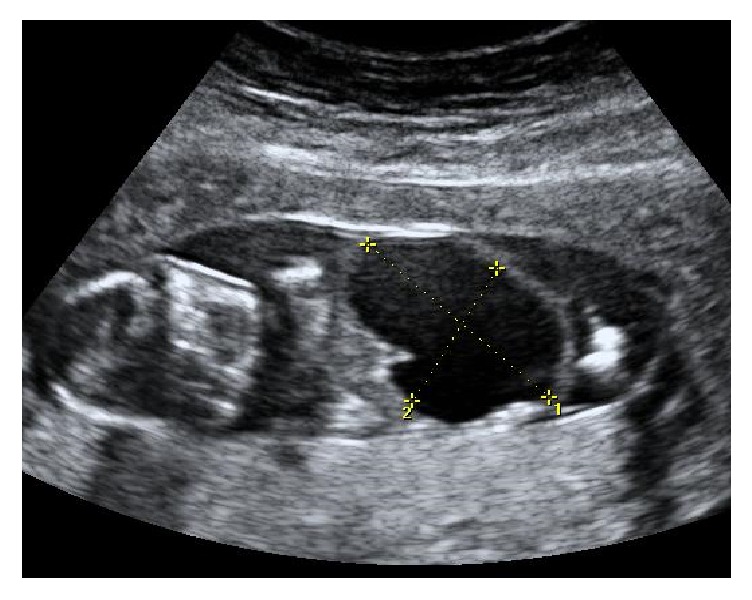
Ultrasonographic findings of the fetus at 14 weeks of gestation. A fetal intra-abdominal cyst (34 × 24 mm in size) and oligohydramnios are observed.

A 40-year-old pregnant woman, gravida 1 para 0, presented with a fetal abdominal cystic lesion with a diameter of 17 mm by ultrasonography at 13 weeks of gestation and was referred to our hospital at 14 weeks of gestation. This was an in vitro fertilization pregnancy. She had a history of aortic dissection at the age of 31 and underwent aortic valve replacement. Thereafter, spironolactone and carvedilol were continuously administered. She underwent ultrasonography on admission, and a fetal abdominal cyst with a size of 34 × 24 mm and oligohydramnios were found (Figure 1). The fetal bladder and bilateral kidneys were not visualized. Also, a single blood vessel running from the aorta anteriorly along the abdominal mass and coursing into the single umbilical artery was seen on color Doppler ultrasonography. Bilateral iliac arteries were not identified. At 17 weeks of gestation, the abdominal mass was enlarged to 46 × 16 mm in size and an amniotic cavity could barely be seen. Precise ultrasonographic evaluation of the fetal lower limbs was limited due to the severe oligohydramnios; the bilateral kidneys and normal bladder were not depicted. We suspected fetal gastrointestinal or urinary tract malformation.

**Figure 2:**
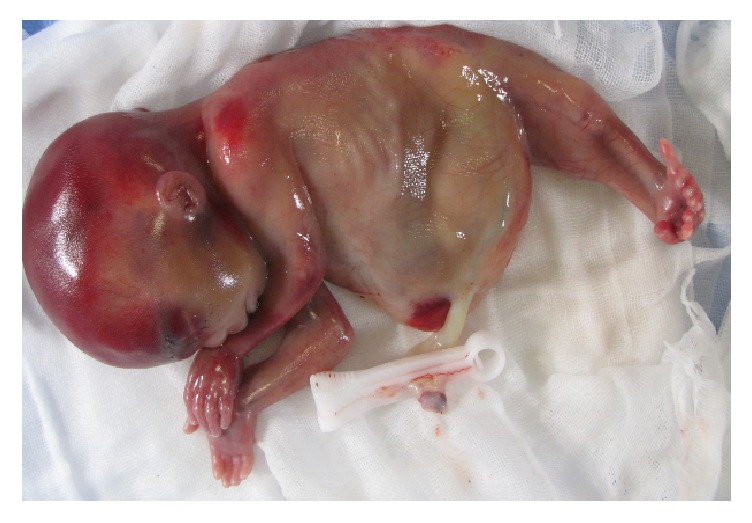
Sirenomelia with fused lower limbs and a bulging abdomen.

Fetal therapy was difficult in this case and the infant's prognosis was considered as extremely poor due to the early severe oligohydramnios. The patient and her husband chose a termination of pregnancy. A fetus weighing 226 g was delivered vaginally at 19 weeks of gestation. The fetus showed fused lower limbs, a bulging abdomen, and absent external genitalia (Figure 2). Two femurs, two tibias, and absent fibulae were found on inspection and palpation of the fused lower limbs, so we arrived at a diagnosis of type III sirenomelia on the Stocker and Heifetz classification [4].

**Figure 3:**
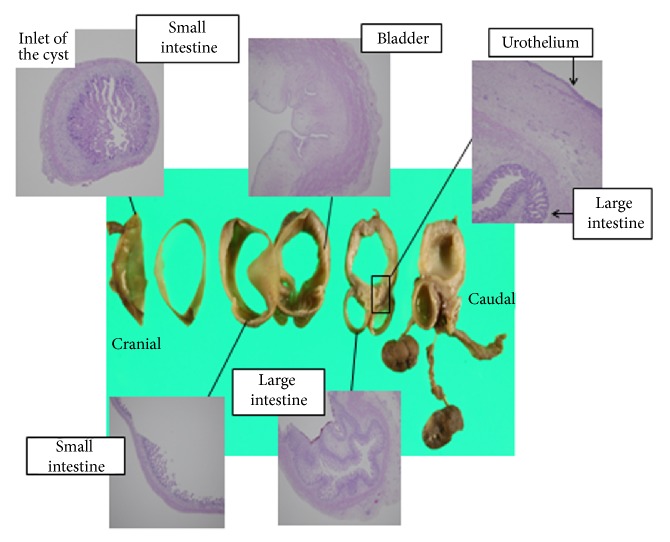
Sections of the specimen of the fetal abdominal cyst and histologic findings (hematoxylin and eosin staining, ×40).

The parents agreed to an autopsy, which revealed a normal stomach, liver, pancreas, and left kidney. Malformations were duodenal atresia, anorectal atresia, and agenesis of the right kidney. The abdominal cavity was occupied by the intra-abdominal cyst, which included clear serous fluid. Histopathological examination revealed that the cyst comprised small intestine epithelium in the anterior region and bladder epithelium and urothelium in the posterior, which turned out to be a saccular cloaca (Figure 3). Fetal karyotyping by chorionic villus sampling resulted in a 46,XY/46,XY del(10)(q11.1) mosaicism fetus.

## 3. Discussion

Infants with sirenomelia are born at an approximate rate of 1 in 100,000 births. Twinning, maternal diabetes, and maternal age less than 20 years are reported as risk factors [5–7]. Maternal exposure to some drugs during pregnancy is a risk factor for fetal sirenomelia. Associations of vitamin A and cocaine with fetal sirenomelia have been reported [8, 9]. Our patient took spironolactone and carvedilol during early pregnancy due to her history of aortic dissection. In rats treated with a high dose of spironolactone, feminization of male rat fetuses was observed [10]. One case report described ambiguous genitalia in a human newborn of a mother treated with spironolactone during pregnancy [11]. However, no association with sirenomelia is known for the two drugs given to our case.

The etiology of the sirenomelia is still unknown. Stevenson et al. [1] advocate “vascular steal theory” as one option. In a persistent vitelline artery, the blood supply for caudal development of the fetus is directed to the placenta, causing caudal blastodermal hypoperfusion. As a result, renal agenesis, bladder and ureteral hypoplasia, deficiency of the genitalia, intestinal dysplasia, anorectal atresia, synsacrum hypoplasia, and malformations of lower limbs are thought to occur.

One of the most consistent vascular features of sirenomelia is the presence of an aberrant umbilical artery derived from a persistent vitelline artery. In our case, an aberrant vitelline artery that developed from the aorta ran alongside the intra-abdominal mass, coursing into the single umbilical artery. It is sufficient for a diagnosis of sirenomelia to depict a persistent vitelline artery and its continuation by using color and power Doppler imaging [2, 12].

There are a few reports of sirenomelia associated with an intra-abdominal cyst. Schiesser et al. [13] reported a case of sirenomelia detected in the first trimester and associated with a fetal intra-abdominal cystic structure. They supposed the cystic lesion to be an expression of the complex malformation of the lower abdomen or the sonographic appearance of necrosis but did not examine the histology of the cyst. Van Keirsbilck et al. [14] reported a case of fetal sirenomelia with a cystic pelvic structure shown by MRI at 14 weeks of gestation, which originated from a dilated sigmoid colon associated with rectal atresia.

In the pathologic findings of our case, the fetal intra-abdominal cyst was found to be comprised of small intestinal epithelium in the anterior region and bladder epithelium in the posterior region and turned out to be a saccular cloaca. In viviparity, at 5 weeks, the midgut and allantoic membrane, as primordia of the small intestine and bladder, form a continued alveus with the hindgut and cloaca. In this case, the fetal intra-abdominal cyst was thought to be originating from this immature structure. There are few reports of histological examination of intra-abdominal cysts in sirenomelia.

It has been reported that ultrasonographic diagnosis of fetal sirenomelia is feasible during the first trimester because of the usually normal amniotic fluid volume, but in the second trimester it becomes difficult due to severe oligohydramnios associated with renal agenesis, resulting in poor depiction of the lower limbs [14, 15]. Oligohydramnios, an aberrant vitelline artery leading to a single umbilical artery, and a fetal abdominal cyst that occupies most of the abdominal cavity in the second trimester are considered as fetal sirenomelia associated with large defects of the gastrointestinal and genitourinary tracts, and the fetus is more likely to be incompatible with life.
